# Human Breast Tissue Microbiota Reveals Unique Microbial Signatures that Correlate with Prognostic Features in Adult Ethiopian Women with Breast Cancer

**DOI:** 10.3390/cancers15194893

**Published:** 2023-10-09

**Authors:** Zelalem Desalegn, Alana Smith, Meron Yohannes, Xueyuan Cao, Endale Anberber, Yonas Bekuretsion, Mathewos Assefa, Marcus Bauer, Martina Vetter, Eva Johanna Kantelhardt, Tamrat Abebe, Athena Starlard-Davenport

**Affiliations:** 1 Department of Microbiology, Immunology, and Parasitology, School of Medicine, College of Health Sciences Addis Ababa University, Addis Ababa 9086, Ethiopia; zelalem.desalegn@aau.edu.et (Z.D.); meron.yohannes@aau.edu.et (M.Y.); tamrat.abebe@aau.edu.et (T.A.); 2 Global Health Working Group, Martin Luther University Halle-Wittenberg, 06097 Halle (Saale), Germany; eva.kantelhardt@uk-halle.de; 3 Department of Genetics, Genomics and Informatics, College of Medicine, University of Tennessee Health Science Center, Memphis, TN 38163, USA; aantoin1@uthsc.edu; 4 School of Medical Laboratory Sciences, Addis Ababa University, Addis Ababa 9086, Ethiopia; meron.yohannes@aau.edu.et; 5 Department of Health Promotion and Disease Prevention, College of Nursing, University of Tennessee Health Science Center, Memphis, TN 38163, USA; xcao12@uthsc.edu; 6 Department of Surgery, School of Medicine, Addis Ababa University, Addis Ababa 9086, Ethiopia; endale.anberber@aau.edu.et; 7 Department of Pathology, School of Medicine, Addis Ababa University, Addis Ababa 9086, Ethiopia; yonas.bekuretsion@aau.edu.et; 8 Department of Oncology, School of Medicine, Addis Ababa University, Addis Ababa 9086, Ethiopia; mathewos.assefa@aau.edu.et; 9 Institute of Pathology, Martin Luther University Halle-Wittenberg, 06097 Halle (Saale), Germany; marcus.bauer@uk-halle.de; 10 Department of Gynecology, Martin Luther University Halle-Wittenberg, 06097 Halle (Saale), Germany; martina.vetter@uk-halle.de; 11 Institute of Medical Epidemiology, Biostatistics, and Informatics, Martin Luther University Halle-Wittenberg, 06097 Halle (Saale), Germany

**Keywords:** breast microbiome, microbiota, Ethiopia, African ancestry, BC, PAM50 molecular intrinsic subtypes

## Abstract

**Simple Summary:**

Breast cancer (BC) is the leading cause of cancer deaths among adult women in Ethiopia. The death toll associated with breast cancer is high among women of African ancestry. The cause of the disparity in mortality is unclear. Recently, studies conducted in the United States and other high-income countries highlighted the role of microbial dysbiosis in breast cancer initiation, growth and treatment outcome. However, whether differences in abundance and composition of microbiota are associated with clinical and histopathological parameters in Ethiopian women has not been studied. The aim of our study was to conduct microbial profiling on breast tumor and normal adjacent tissues of the same donor. Further, the study aimed to identify the association of the differences in microbial composition and abundance with clinicopathological factors in Ethiopian women with breast cancer. This is the first study to report an association between breast microbial dysbiosis and clinicopathological factors in Ethiopian women.

**Abstract:**

Breast cancer (BC) is the leading cause of cancer mortality among women in Ethiopia. Overall, women of African ancestry have the highest death toll due to BC compared to other racial/ethnic groups. The cause of the disparity in mortality is unclear. Recently, studies conducted in the United States and other high-income countries highlighted the role of microbial dysbiosis in BC initiation, tumor growth, and treatment outcome. However, the extent to which inter-individual differences in the makeup of microbiota are associated with clinical and histopathological outcomes in Ethiopian women has not been studied. The goal of our study was to profile the microbiome in breast tumor and normal adjacent to tumor (NAT) tissues of the same donor and to identify associations between microbial composition and abundance and clinicopathological factors in Ethiopian women with BC. We identified 14 microbiota genera in breast tumor tissues that were distinct from NAT tissues, of which *Sphingobium*, *Anaerococcus*, *Corynebacterium*, *Delftia*, and *Enhydrobacter* were most significantly decreased in breast tumors compared to NAT tissues. Several microbial genera significantly differed by clinicopathological factors in Ethiopian women with BC. Specifically, the genus *Burkholderia* more strongly correlated with aggressive triple negative (TNBC) and basal-like breast tumors. The genera *Alkanindiges*, *Anoxybacillus*, *Leifsonia*, and *Exiguobacterium* most strongly correlated with HER2-E tumors. Luminal A and luminal B tumors also correlated with *Anoxybacillus* but not as strongly as HER2−E tumors. A relatively higher abundance of the genus *Citrobacter* most significantly correlated with advanced-stage breast tumors compared to early-stage tumors. This is the first study to report an association between breast microbial dysbiosis and clinicopathological factors in Ethiopian women.

## 1. Introduction

Globally, female breast cancer (BC) is becoming the most common malignancy [1]. In Ethiopia, it is the leading cause of cancer-morbidity, attributable to one-third of the total cancers occurring in women [2,3]. Annually, the incidence of BC accounts for a total of 16,133 cases and approximately 9000 deaths in Ethiopia [4]. It has been shown that more than 80% of cancer cases in Ethiopia are identified at an advanced stage, which has been attributed to a lack of resources necessary for early BC detection and prevention strategies [5]. Though there are additional unforeseen factors contributing to BC risk, the increasing incidence of BC in sub-Saharan Africa may be associated with a dynamic change in lifestyle behaviors, reproductive factors, and population aging [6,7,8]. 

Recently, scientific evidence demonstrated that microbial dysbiosis in the breast microenvironment may contribute to BC development [9,10,11,12,13]. Consequently, microbiota inhabiting breast tissue and/or the tumor microenvironment (TME) have a potential biological function in mediating carcinogenesis in breast tissue [10,14]. Variations in composition and functionality of the microbiota among BC cases in relation to healthy controls encourage the potential development of microbiome-derived biomarkers and future targeted interventions which ultimately have a central role in reducing the burden of BC [15]. 

Indeed, a combination of genetic, environmental, and lifestyle factors have been linked to BC [14], in which bacterial communities within the host could be one additional environmental factor associated with BC [16]. In general, supporting evidence from epidemiological studies confirms that various microbial species and/or their metabolites contribute to at least 16% of malignancies that occur across the globe [17]. However, it is unclear as to how microbial dysbiosis contributes to BC development. It is hypothesized that microbiota may utilize different mechanisms to promote cancer development, such as modulating inflammation via mediators and inflammatory response signaling pathways [18], triggering DNA damage [19], and/or releasing harmful gut microbiota-derived metabolites that mediate tumorigenesis or tumor suppression [20,21].

Regardless of factors including sample collection site, age of patients with BC, geographical variation, history of pregnancy, presence/absence of breast malignancy, method of DNA preparation, and sequencing technologies, the composition of mammary microbiota appeared diverse and different when compared with other body sites [13,22]. The unique breast microbiota pattern of healthy women demonstrated that the predominant phyla were Proteobacteria, Firmicutes, Actinobacteria, and Bacteriodetes in decreasing order, respectively. Though evaluation of microbiota in breast tumor tissue has recently received much attention, previous research works highlighted distinct microbiota in human milk which has already been established for several years. Similarity in composition between human milk and breast tissue microbiota were noted, and Proteobacteria was identified as the most abundant phylum [18,19]. 

Furthermore, tumor microenvironment (TME) has become an important focus in understanding BC development and progression, and in determining responses to treatment over the last couple of decades [23]. The breast tumor microbiome has been shown to play an important role in modifying the TME, thereby potentially impacting treatment outcome [24]. In fact, it is recognized that the TME is an integral component when investigating the tumor microbiome [25]. This is further highlighted in recent studies that observed differences in breast microbial composition and density between NAT and breast tumor tissues and/or healthy controls [12,26]. 

Although the microbiomes of populations from developed countries have been extensively characterized, evidence-based data using African populations to address this topic remain limited [12,27,28,29], yet collecting such data has to be a high priority. Furthermore, to our knowledge, no microbiome-based BC studies have been conducted in Ethiopian women. It is critical for breast microbiome studies to be replicated in Africa due to inter-individual and population variability in cultural behaviors, environmental exposures, including infectious diseases, and genomic heterogeneity [30,31,32,33,34]. Therefore, this study was carried out to investigate microbiota abundance among patients with BC and explore its correlation with clinical and histopathological features in Ethiopian women with BC. 

## 2. Materials and Methods

### 2.1. Patient Enrollment and Tissue Collection and Processing

Pathologically confirmed BC patients (age 16 to 83) were enrolled into the study from select hospitals in Addis Ababa, Ethiopia, according to the set inclusion and exclusion criteria. All study participants who were taking antibiotics were excluded from this study. BC patients were approached by physicians while attending for routine services and informed about the objective and procedures of data collection. After securing the written informed consent, we applied aseptic procedures and employed a standard protocol to obtain a fresh frozen surgically resected breast tissue specimen from patients with BC who underwent mastectomy. The specimens constituted a total of 100 surgically obtained tissues composed of breast tumor (T) tissue and normal adjacent tissue (NAT) pairs from the same donor (*n* = 50) and were included in this study for comparison from women of Ethiopian descent. Normal breast tissue immediately adjacent (up to 5 cm) to the collected breast tissue sample was evaluated and confirmed by a pathologist to be histologically free of any tumor cells or lesions. Upon receipt, breast tissue samples (*n* = 100) were immediately transferred to a sterile container containing liquid nitrogen and maintained at −80 °C until further processing. Clinical and histopathological data about the donor breast tissue specimen, including hormone receptor (HR) status, Ki-67 proliferation index, tumor grade, and stage of BC was obtained via medical chart review and pathological evaluation. This study was carried out according to the Declaration of Helsinki, and the research protocol was ethically approved by the Institutional Review Board of the College of Health Sciences, Addis Ababa University (IRB protocol #092/17/17), and at the University of Tennessee Health Science Center (IRB #19-06743). 

### 2.2. Immunohistochemistry (IHC) and Gene Expression Analysis

Samples of the fresh frozen tissues were embedded and analyzed for the expression of estrogen receptor (ER), progesterone receptor (PR), human epidermal growth factor receptor 2 (HER2), and Ki-67 proliferation index using immunohistochemistry (IHC). IHC analyses was carried out using specific antibodies directed against the respective proteins as presented in Table 1.

Following the IHC analysis, the immune reactive score reading was recorded and the status of ER and PR expression interpreted according to established guidelines [35].

In general, HR positivity was declared with IRS > 0 whereas negativity was defined as IRS < 1. HR status was declared positive when either ER and/or PR tended to be positive. ASC-CAP guidelines were employed to assess the status of HER2 [36]. On the other hand, the Ki-67 proliferation index was scored as follows:

- ‘Low proliferation index:’ when Ki-67 staining was positive in <20% of tumor cells; or
- ‘High proliferation index’: when at least 20% of tumor cells stained positive [37,38].

Histological grading was performed using the Elston–Ellis grading system [39].

### 2.3. RNA Extraction, Gene Expression Profiling, Normalization, and Intrinsic Subtyping

#### 2.3.1. RNA Extraction

First the pathologist identified the tumor-enriched area on HE-stained slides. Then, multiple sections consisting of 10 µm breast tissue were utilized for RNA extraction. RNA was extracted using the miRNeasy FFPE Mini Kit® (Qiagen) following the manufacturer’s recommendations. Before and after extraction, we decontaminated the workbench with disinfectant and chemicals to ensure a nuclease-free environment and to maintain the quality of quality of RNA extraction and further downstream applications. After measuring the concentration and the quality using a nanophotometer, the extracted RNA was stored at −80 °C for further downstream applications.

#### 2.3.2. NanoString and PAM50 Assay

To determine intrinsic subtypes, a NanoString nCounter^®^ Analysis System (NanoString Technologies, Seattle, WA, USA) was employed. We measured the relative gene expression using the PAM50 method, a multiplexed hybridization assay, where digital readouts of fluorescent barcoded probes hybridize with each mRNA sequence of interest. nCounter^®^ Digital Analyzer was used for the acquisition of data. Importing data, verifying quality control, and normalization of expression levels were performed using nSolver software version 4. Furthermore, negative input controls were utilized for background subtraction from raw transcript counts. A total of six reference-control genes were used to perform reference-normalization by dividing the geometric mean of these genes. Thereafter, the normalized mRNA counts were log2-transformed. Finally, the distinct intrinsic subtype classification was estimated using the nearest PAM50 centroid algorithm in Bioclassifier and NanoStringNorm implemented in R [40] and according to the classification method as described previously [41].

### 2.4. DNA Extraction, PCR, and 16S rRNA Gene Sequencing and Processing

Using aseptic techniques, we isolated DNA from breast tissues under a sterile laminar flow hood using the Qiagen DNA Isolation kit (Qiagen, Germantown, MD, USA). We decontaminated the workbench with disinfectant and chemicals to ensure a nuclease-free environment. DNA quality and quantity was measured using the Nanodrop and a Quant-iT PicoGreen dsDNA Assay Kit (Invitrogen, Carlsbad, CA, USA). DNA samples were then shipped off on dry ice to Microbiome Insights (Vancouver, BC, Canada) for further processing. Specimens were gently placed into a MoBio PowerMag Soil DNA Isolation Bead Plate, which is needed for further extraction of microbial DNA and for the isolation of nucleic acids without the binding of residual contaminants and purified on a KingFisher robot following MoBio’s instructions. Bacterial 16S rRNA genes were PCR-amplified using dual-barcoded primers that target the V4 region of the bacterial genome, as per the protocol of Kozich et al. [42]. Amplicons were further sequenced using the 300-bp paired-end kit (v.2) on an Illumina MiSeq instrument. The potential for contamination was addressed by co-sequencing DNA amplified from specimens and from template-free negative controls and DNA extraction kit reagents processed the same way as the specimens. A cloned SUP05 DNA sample served as a positive control. Resulting sequences were filtered, then trimmed and denoised, and used to infer amplicon sequence variants (ASVs) exactly using the DADA2 sequencing data tool [43]. Subsequent ASVs were merged to create a sequence table, then chimeras were removed and taxonomically classified using the Ribosomal Database Project’s Training Set 16 (11.5 release) as the reference database by the naive Bayesian classifier method implemented in DATA2. The Phyloseq program [44] was further used to import, store, analyze, and graphically display complex phylogenetic sequencing data. 

### 2.5. Statistical Analysis

We estimated alpha diversity, defined as the observed richness or number of taxa of each sample, using the Shannon index on raw ASV abundance tables after filtering out all contaminants. The Simpson index is another indicator we used to further estimate microbial diversity and to evaluate species evenness. We conducted a Kruskal–Wallis test or Wilcoxon signed rank test to determine significant differences in diversity. To estimate beta diversity across samples, we excluded ASVs occurring with a count of less than 5 (5% of number of samples). We then computed Bray–Curtis indices. Beta diversity, defined as the variability in community composition or identity of taxa observed among the samples, was visualized using principal coordinate analysis (PCoA) ordination and emphasized differences across samples. Variation in community structure was assessed with permutational multivariate analysis of variance (PERMANOVA) with groups/subtypes as the main fixed factor and using 999 permutations for significance testing [45]. All analyses were conducted in the R environment.

## 3. Results

### 3.1. Clinicopathological Characteristics of the Study Participants

This study aimed to enhance our understanding of breast microbial abundance and composition in Ethiopian women with BC. Our study included fresh frozen tumor tissue and normal adjacent to tumor (NAT) specimens from 50 Ethiopian women diagnosed with BC, for a total of 100 breast tissue samples analyzed. Table 2 presents the clinicopathological characteristics of the study participants. The mean age of patients in this study was 46 ± 1.92 years. Of these women, 60% were premenopausal, 50% were with confirmed early stage at the time of diagnosis, and almost equal proportions of the tumors were intermediate to poorly differentiated (G2, G3). With regards to their IHC groups, 44 (88%) were HR+. Of the breast tumor tissues, 26% were luminal A, and 17% were luminal B.

### 3.2. Breast Tumor Tissue Exhibits Distinct Microbiome Composition from NAT Tissue of the Same Woman

In this study, we were able to sequence the 16s rRNA amplicon in 50 paired samples from tumor tissue and in NAT tissue. We sequenced 16Sv4 amplicons generated from human breast tissue samples and minimized the potential of bacterial contaminants by using multiple controls alongside the tissue samples. After quality-filtering and inferring amplicon sequence variants (ASVs), quality-filtered reads accounting for an average of 1569 were generated per sample and used for further analysis. Rarefaction curves relating number of sequencing reads compared to the number of ASVs or genera are shown in Figure S1A,B.

**Figure 1:**
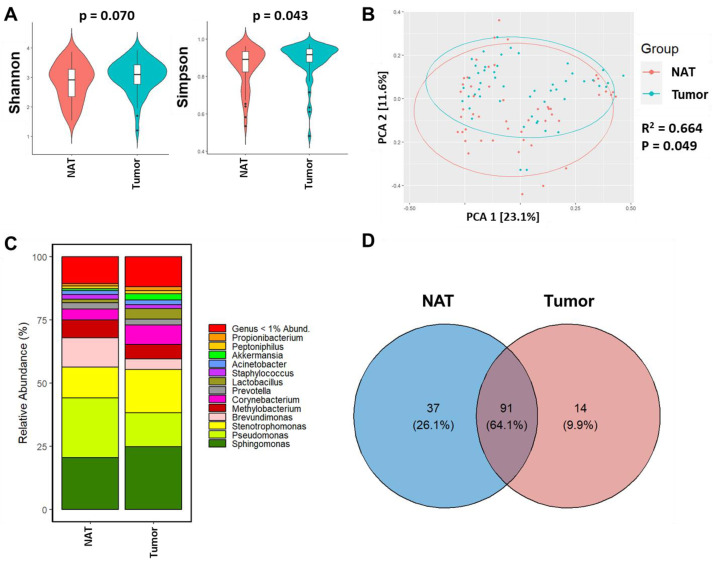
Breast bacterial community composition varies by patient BC status and normal adjacent tumor (NAT) and tumor tissue types. (**A**) Violin plots show median and interquartile range in bacterial alpha diversity as measured by Shannon and Simpson diversity indices within (tumor and NAT) breast tissue from Ethiopian BC patients. *p*-values were obtained from Kruskal–Wallis tests. (**B**) Principal coordinates (PC) plots show the clustering pattern of tumor and NAT based on unweighted UniFrac distance and are colored by sample types (red circles—NAT, teal—Tumor samples); *p* = 0.049 and R^2^ = 0.664. (**C**) Taxonomic composition of the breast microbiome depicted as proportional abundances at the genus level for NAT and corresponding tumor tissue of the same donor. (**D**) Venn diagram showing the unique and shared bacterial genera between the tumor and normal groups.

In this study, alpha diversity was quantified to correlate the differences in breast microbial diversity between the tumor and NAT breast tissues. Interestingly, the Shannon index showed alpha diversity was not significantly different between tumor and NAT tissues (*p* = 0.07) (Figure 1A); however, alpha diversity measured by the Simpson index revealed slightly higher alpha diversity in tumors compared to NAT (*p* = 0.043). To determine differences in beta diversity, we visualized the overall differences between the breast microbiome profile of tumor and NAT tissues using principal coordinate analysis (PCoA) of Bray–Curtis dissimilarity (Figure 1B). The tumor tissue and NAT clustered significantly differently between the two groups (R^2^ = 0.664; *p* = 0.049) with greater dissimilarity along PC1 (23.1% variation). We also observed overlap between the two groups. 

We then determined the taxonomic composition and abundance of breast microbial profiles of tumor and NAT tissues. The 16S rRNA based sequencing identified 5 phyla, 7 classes, 16 orders, 16 families, and 16 genera across all the breast tissue samples. Across all tissue types, the three predominant phyla were Proteobacteria (48.4%), followed by Firmicutes (22.1%) and Actinobacteria (15.0%) as shown in Figure S2A. Alphaproteobacteria (41.5%), Bacilli (15.2%), Actinobacteria (15.0%), and Bacteroidia (7.33%) were the most abundant classes among all tumor and NAT tissue samples (Figure S2B). Considering all breast tissue samples, the most prevalent families were *Sphingomonadaceae* (20.78%), *Staphylococcaceae* (13.2%), *Beijerinckiaceae* (13.1%), and *Corynebacteriaceae* (9.44%) (Figure S2C). We also observed that 64.1% of microbial genera were shared between NAT and tumor groups. However, 26.1% of genera were unique to NAT tissues, whereas approximately 10% were unique to tumors (Figure 1D). Genera most predominant in tumor compared to NAT tissues included *Sphingobium* (*p* < 0.0001), *Anaerococcus* (*p* < 0.0001), *Corynebacterium* (*p* = 0.0012), and *Delftia* (*p* = 0.0031) (Table S1). 

### 3.3. Breast Microbial Communities Differ by Breast Tumor IHC Types, PAM50 Intrinsic Subtypes, and Stage of Disease

**Figure 2:**
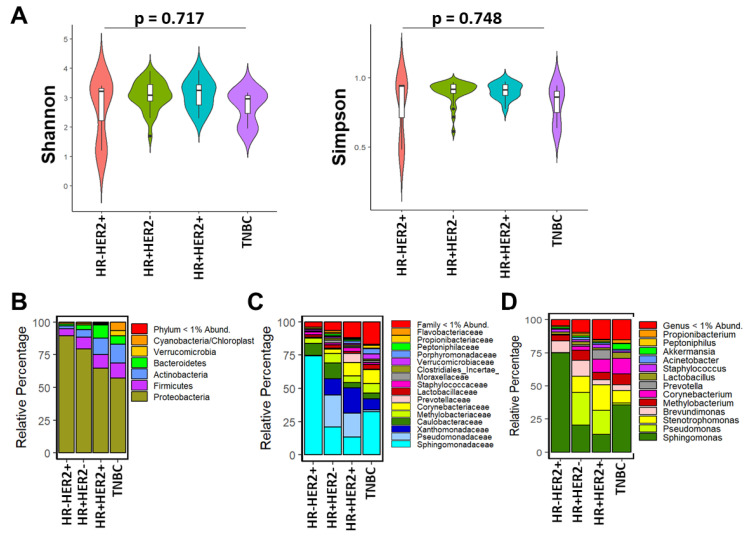
Breast bacterial community composition varies by immunohistochemical (IHC) status. (**A**) Violin plots show median and interquartile range as measured by Shannon and Simpson diversity indices with regard to hormone receptor (HR) and HER2 status of the tumor tissue. *p*-value results were from Kruskal–Wallis tests. Taxonomic composition of the breast microbiome is depicted as relative percentages at the (**B**) phylum, (**C**) family, and (**D**) genus level.

We next determined whether microbial composition and abundance were associated with breast tumor prognostic features, including by IHC group and PAM50-based intrinsic subtypes. The Shannon index (*p* = 0.717) and Simpson index (*p* = 0.748) did not differ significantly by HR status, respectively (Figure 2A). Proteobacteria was the predominant microbiota observed followed by Firmicutes (Figure 2B). The predominant bacterial phylum, family, and genus across the four IHC breast tumor groups were Proteobacteria, *Sphingomonadaceae*, and *Sphingomonas*, respectively, as shown in Figure 2B–D. The relative abundances of these phyla differed by HR status, with TNBC tumors having the lowest abundance of Proteobacteria compared to the other tumor types. Microbial composition at the lower taxonomic level (family and genus) were more abundant and/or diverse in HR+ HER- and HR + HER2+ tumors compared to HR− and TNBC. We did further analysis to understand differences in relative abundances of the bacteria at the genus level among the different IHC types using a linear model. We found that *Exiguobacterium* (*p* = 0.007), *Varibaculum* (*p* = 0.0087), and *Leifsonia* (*p* = 0.0016) were significantly less abundant in HR+/HER2−, HR+/HER2+ and TNBC tissues compared to HR − HER2+ tumors (Table S2).

**Figure 3:**
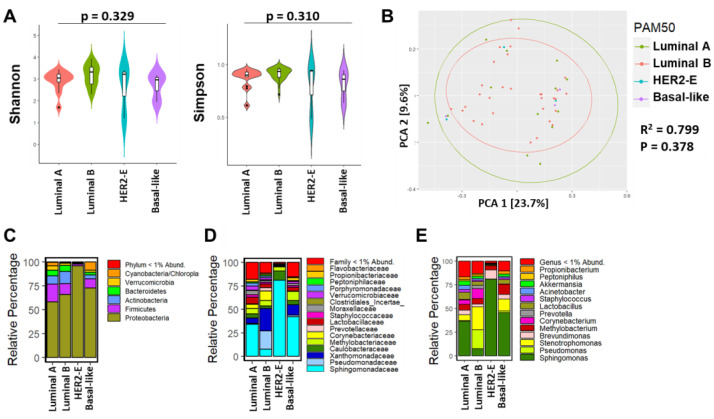
Breast bacterial community composition varies by PAM50 intrinsic subtypes. (**A**) Violin plots show median and interquartile range as measured by Shannon and Simpson diversity indices within breast tissue from Ethiopian BC patients. *p*-value results were obtained from Kruskal–Wallis tests. (**B**) PC plot shows the clustering pattern of the intrinsic subtypes based on unweighted UniFrac distance and is colored by sample types (green—luminal A, red circles—luminal B, teal—HER2E, and purple circles—basal-like tumor samples); *p* = 0.378 and R^2^ = 0.799. Taxonomic composition of the breast microbiome, depicted as relative percentage at the (**C**) phylum, (**D**) family, and (**E**) genus level.

Since we observed significant differences in the relative percentage of microbiota according to HR status, we hypothesized that microbial differences exist across intrinsic subtypes. Similar to IHC type, Shannon and Simpson indexes were not significantly different between the four intrinsic subtypes (*p*-value = 0.329 vs. *p*-value = 0.310, respectively) (Figure 3A), which was also observed among the PCA analysis (R^2^ = 0.779, *p* = 0.378) (Figure 3B). Furthermore, Proteobacteria were the predominant bacterial phylum across the four intrinsic breast tumor subtypes followed by Firmicutes (Figure 3C), whereas at the family level, *Sphingomonadaceae* were predominant in luminal A, HER2-E and basal-like breast tumor intrinsic subtypes (Figure 3D). In the luminal B subtype, the relative abundance of such microbiota was lower, including *Sphingomonadaceae*; however, *Pseudomonadaceae* and *Xanthomonadaceae* were relatively higher (Figure 3D). At the lower taxonomic level (genus), our linear regression analysis revealed differences in abundance of microbial genera by intrinsic subtype. As shown in Figure 3E, statistically significant abundance of *Polaromonas* (*p* = 0.0005), *Varibaculum* (*p* = 0.0087), *Exiguobacterium* (*p* = 0.0097), *Bifidobacterium* (*p* = 0.0382), and several other genera between luminal A, HER2-E, and basal-like tumors were observed overall when compared to luminal B tumors (Table S3). 

**Figure 4:**
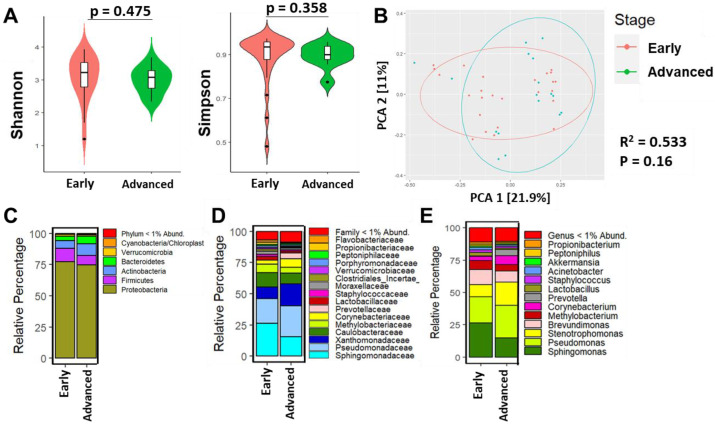
Breast bacterial community composition varies between early and advanced stages of BC. (**A**) Violin plots show median and interquartile range as measured by Shannon and Simpson diversity indices based upon breast tumor stage. *p*-value results were obtained from Kruskal–Wallis tests. (**B**) PC plots show the clustering pattern among early (red circles), advanced (green circles), and unknown (blue circles) tumor stage based on unweighted UniFrac distance; *p* = 0.16 and R^2^ = 0.533. (**B**) Taxonomic composition of the breast microbiome, depicted as relative percentage at the (**C**) phylum, (**D**) family, and (**E**) genus level.

We next investigated the relationship between tumor stage and the relative abundances among microbial taxa. Shannon (*p* = 0.475) and Simpson (*p* = 0.358) indexes were not significantly different between early and advanced stage of disease, respectively (Figure 4A). PCA showed no significant separation between early and advanced stages of BC (R^2^ = 0.533, *p* = 0.16). However, visually there is a clear separation between the two groups. There were eight patients with unknown stage information which did not significantly change the results (Figure 4B). At the phylum level, Proteobacteria dominated (Figure 4B). Similar to HR status and intrinsic subtype, relative abundance of family *Sphingomonadaceae* and genus *Sphingomonas* dominated in early-stage (T0-2A) compared to more advanced-stage tumors (T2B-4) (Figure 4C,D). Specifically, genus *Stenotrophomonas* (*p* = 0.015), *Corynebacterium* (*p* = 0.049), *Prevotella* (*p* = 0.024), *Actinomyces* (*p* = 0.021), and several additional genera were significantly more abundant in advanced-staged tumors compared to early-stage tumors (Table S4). 

### 3.4. Breast Microbial Communities Correlate with Clinicopathological Features in BC

**Figure 5:**
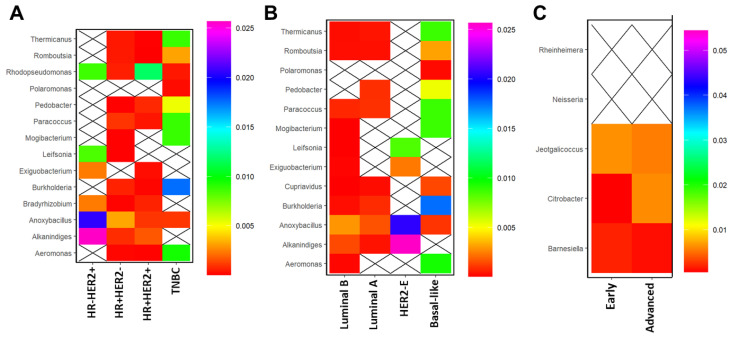
Specific bacterial genera correlate with clinicopathologic features. Mean relative abundances (proportions) of bacterial genera that were differentially present in breast tumors stratified by (**A**) IHC type (**B**) PAM50 breast tumor subtype, and (**C**) early or advanced stage. Crossed-out boxes indicate samples for which specific genera were not detected. Color bars vary on a logarithmic scale. All genera shown had FDR-corrected *p*-value < 0.05 by Kruskal–Wallis H-test.

Lastly, we determined whether microbial communities correlated with certain clinicopathological features, including IHC group, intrinsic subtype, and stage, as observed in our study. In HR−/HER2+ BC, *Alkanindiges* and *Anoxybacillus* strongly correlated with this IHC group, whereas *Rhodopseudomonas* correlated strongly with HR+ HER2+ BC. Only the genus *Anoxybacillus* slightly correlated positively with HR + HER2- tumors. Interestingly, TNBC tumors correlated strongly with the genus *Burkholderia*, followed by *Thermicanus*, *Paracoccus*, *Mogibacterium*, and *Aeromonas* (Figure 5A). 

Many of the same microbial patterns observed by IHC group were also found among the different intrinsic molecular subtypes. For instance, similar to HER2+ tumors, HER2-E tumors correlated strongly with *Alkanindiges* and *Anoxybacillus* (Figure 5B). Likewise, basal-like tumors correlated with the same genera as TNBC, with *Burkholderia* being the most strongly correlated genus. There was a weak association with *Cuprivadus* in basal-like tumors (Figure 5B) that was not observed in TNBC tumors using IHC determination (Figure 5A). Luminal A and luminal B tumors correlated weakly with *Anoxybacillus*. Furthermore, there was not much of a difference in genera among the early- and advanced-stage BC, although a stronger correlation with the genus *Citrobacter* was observed in advanced breast tumors (Figure 5C). 

In summary, we identified 14 microbiota genera in breast tumor tissues that were distinct from NAT tissues, of which *Sphingobium*, *Anaerococcus*, *Corynebacterium*, *Delftia*, and *Enhydrobacter* were most significantly decreased in breast tumors compared to NAT tissues. Several microbial genera also significantly differed by clinicopathological factors in Ethiopian women with BC. Specifically, genera *Burkholderia*, followed by *Thermicanus*, *Paracoccus*, *Mogibacterium*, and *Aeromonas*, more strongly correlated with aggressive triple negative (TNBC) and basal-like breast tumors compared to less aggressive luminal A, luminal B, and HER2-E tumors. The genera *Alkanindiges*, *Anoxybacillus*, *Leifsonia*, and *Exiguobacterium* most strongly correlated with HER2-E tumors. Luminal A and luminal B tumors also correlated with *Anoxybacillus* but not as strongly as HER2-E tumors. A relative higher abundance of the genus *Citrobacter* most significantly correlated with advanced-stage breast tumors compared to early-stage tumors. This is the first study to report an association between breast microbial dysbiosis and clinicopathological factors in Ethiopian women.

## 4. Discussion

Women in Ethiopia, like women in other lower middle income countries (LMIC), have poorer overall BC survival rates [46,47]. Stage at diagnosis as well as availability of optimal care were among the factors associated with poor BC survival outcomes. Additionally, the role of biological factors including the tumor microenvironment (TME) that includes the microbiota have been shown to be associated with poor clinical outcomes [48].

BC is a leading cause of cancer morbidity among women of African ancestry. This is further compounded by underrepresentation of women of African ancestry in BC research studies. Therefore, we intended this study to generate evidence-based preliminary data to profile the microbiome in breast tumor and normal adjacent tissues of the same donor and to identify the association between differences in microbial composition and abundance and clinicopathological factors in Ethiopian women with BC. To this end, we used fresh frozen breast tumor and NAT tissues to profile microbial composition and abundance and to determine their association with clinicopathological features observed in BC.

### 4.1. Findings of Studies on Breast Tissue Microbiome

In recent years, research findings have begun to reveal the potential role of mammary microbiota in mediating BC development [49]. Current research findings indicate that the microbiota among BC patients differs from that of healthy women [14]. Supporting evidence reported a distinct mammary microbiota composition that also differed between breast tumor and normal breast tissues [11]. Such evidence points to distinct microbial signatures that might be related to cancer development and response to certain treatment [14]. However, the extent to which microbiota alterations (dysbiosis) contributes to BC development is unclear. Although the discovery of microbiota differences in breast milk was previously explored, recent works have begun to investigate the association between breast tissue microbial dysbiosis with cancer. Accordingly, these efforts indicate that healthy breast and BC tissues are composed of a unique microbiota in which the phylum Proteobacteria predominated in healthy breast tissues, followed by Firmicutes [11,50]. 

In agreement with our study, previous studies demonstrated that the breast is characterized by a high predominance of Proteobacteria, followed by the phyla Firmicutes and Actinobacteria [11,49,50]. In another study, Proteobacteria were prominently identified in the tumor tissues and conversely, in non-cancerous adjacent tissues, Actinobacteria abundance was increased [51]. Furthermore, previous studies highlighted the difference in breast microbial composition and abundance between normal adjacent and tumor tissues and/or healthy controls [12,26]. These microbes thrive in a fatty-acid-rich environment in the breast and are positively associated with adiposity [52]. Thus, it is not surprising that these bacteria are abundant in the breast. At the family level, our findings are in agreement with Klann et al., who also observed a higher relative abundance the family *Ruminococcaceae* and the genus *Akkermansia* in breast tumor tissue as compared to normal tissue and a lower relative abundance of *Bacteroides* and *Sutterella* in breast tumor compared to normal tissue [53]. More recently, exciting research on the breast microbiome to identify distinct microbial signatures considering different parameters, including race of patients with BC, tumor stage, and breast tumor subtype, was carried out [28]. In agreement with our findings, it was observed that the phylum Proteobacteria was most abundant across all tissues, followed by Firmicutes, Bacteroidetes, and Actinobacteria regardless of race, and a higher abundance of the genus *Ralstonia*, which could explain in part a portion of the BC racial disparities [28]. Considering the diverse population in the Ethiopian context, conducting a study to profile microbiota and understanding its association with certain prognostic features would be of paramount importance in cancer management. 

In efforts towards understanding the role of microbiota in health and disease, until now, it has remained elusive and/or unclear whether there is a difference in microbial composition in breast tumors and paired NAT of the same individual with BC [11,13]. While comparing tumor tissue with NAT, the microbiota composition showed a distinct bacterial profile, suggesting the oncogenic effect of specific bacterial taxa [50]. In terms of relative distribution of microbiota, our sequencing data of NAT and tumor groups are supported by another study where out of 11 differentially abundant operational taxonomic units (OTUs), the majority of OTUs were abundant in paired normal tissue and close to one-third of the OTUs were abundant in tumor tissue. The bacterium *Sphingomonas yanoikuyae* was enriched in paired normal tissue, similar to our findings, whereas the bacterium *Methylobacterium radiotolerans* was most significantly enriched and prevalent in paired in tumor tissue [13]. Microbial differences in paired breast tissues (tumor versus NAT) have also been identified in women from different geographical locations, which suggests that environment or lifestyle factors might impact the relative abundance of certain microbes in the breast microenvironment [11,54].

Generally, the breast TME is composed of a variety of cell types and microbiota. Studies suggest that pathophysiological changes occurring within the breast cells could have a significant impact on tumor growth [51]. Additionally, it has been discovered that certain microbial species identified in the human breast are potent agents that have the potential to trigger DNA damage, genomic instability, and alterations of molecular events in the form of mutations and epigenetic modifications [55]. For instance, functional in vitro studies revealed that *Escherichia coli* and *Staphylococcus epidermidis* isolated from BC patients trigger DNA double-stranded breaks in HeLa cells [22]. Therefore, microbial communities within a host could be considered an additional environmental factor that may contribute to or be influenced by carcinogenesis [56]. Considering that the data in this regard are in their infancy, precautions are required while inferring the significance of microbiota in the initiation, progression, and prediction of therapeutic responses among patients with BC. 

### 4.2. Findings Associated with Clinical and Histopathological Features

In agreement with our findings, other studies reported a correlation between specific breast microbial taxa and certain prognostic features such as stage [12], receptor status [26,57,58], and lymphovascular invasion [26]. In our study, we found specific genera that were strongly associated with different intrinsic subtypes and by stage of disease. Specifically, we observed that the genus *Burkholderia* most strongly correlated with aggressive triple negative (TNBC) and basal-like breast tumors compared to less aggressive luminal A, luminal B, and HER2-E tumors. In support of our findings, a recent insightful study by Hoskinson et al. noted that a higher abundance of several microbial families, including *Burkholderiaceae*, was associated with early BC development when analyzing normal breast tissue compared to tissues donated prior to and after BC diagnosis [59]. These findings suggest that microbes in the genus *Burkholderia* may play an oncogenic role in the development of aggressive BCs, such as TNBC and basal-like tumors. In addition, we observed that several genera, including *Alkanindiges*, *Anoxybacillus*, *Leifsonia*, and *Exiguobacterium* most strongly correlated with HER2-E tumors. Luminal A and luminal B tumors weakly correlated with *Alkanindiges* and *Anoxybacillus*. Interestingly, Modica and colleagues reported that lymphovascular invasion correlated negatively with *Alkanindiges* in HER2-E tumors, which could explain the weak association between less aggressive luminal A and luminal B tumors in our study [60]. On the other hand, *Alkanindiges* was correlated with invasive ductal carcinomas [61]. Furthermore, we observed a relative higher abundance of the genus *Citrobacter* most significantly correlated with advanced-stage breast tumors compared to early-stage tumors. A supporting study by Yang et al. reported that women with malignant BC exhibited enriched levels of *Citrobacter* in their gut compared to those with benign tumors where *Citrobacter* was further associated with elevated glycan and lipopolysaccharide biosynthesis [62]. 

With a comparatively large sample size aimed at identifying bacterial genera with statistically different abundances, another study identified *Porphyromonas*, *Lacibacter*, *Ezakiella*, and *Fusobacterium* as being more abundant in higher-stage compared with lower-stage tumors [61]. These findings highlight the complex nature of the microbiome and tumor interactions in the breast. Although we did not observe the distribution across tumor grade, in a separate study, multiple genera were significantly associated with histologic grade, with a number of genera present only in grade 1 tumors [61]. However, additional studies using a larger sample size and comparable numbers of different groups of BCs are needed to confirm these associations. Collectively, most findings consistent with our study revealed that the breast houses a unique microbiota that can be distinguished based upon hormone receptor status, molecular subtype, and stage of disease [11,13,50,51]. 

### 4.3. Problems with Breast Microbiome Studies

Normally, a wide range of research findings employ various platforms to investigate breast tissue microbial profiles. Consequently, comparing and interpretating findings from different studies can be challenging due to differences in sample retrieval, processing, and methods employed to prevent bacterial contamination. According to recent evidence, microbiota of BC tissues are quite different from microbiota in breast tissues of women without BC [26]. Though various studies have indicated that breast microbiota might play a role in mediating breast carcinogenesis [11,22,63,64], scientists are posing questions about how the microbiome might play a role in modulating the risk of BC development. The hypothesis is that changes in the composition of breast microbiota may also contribute to disease development and progression through several pathways, but it is still unclear whether the host´s microbial differences are a consequence or a cause of this human disease. This distinct phenomenon could be explained and/or interpreted in two ways: (1) alteration in microbiota profiles and/or dysbiosis comes first in the course of the carcinogenic process and can establish a microenvironment predisposed to cancer or (2) there is no correlation between the two events. However, knowledge of the microbiota of BC patients remains in its infancy. Given this context, additional studies have to be carried out to broaden knowledge on this topic and to understand which of the two possibilities occurs in patients with BC. 

Considering the observed methodological differences in breast microbiome studies, including sample size, sample type, employed amplicon amplification technique, DNA extraction method utilized, sequencing approach, and potentially other factors, appreciating the precise difference in the composition and abundance of microbiota within a group of variables could be challenging in interpreting conclusions. However, in support of our findings, the literature indicates that differences in community composition across data sets can be attributed to ethnicity, dietary habits, geography, lactation status, method of sample collection, and platform of sequencing and data analysis [65]. 

In addition to the contradicting scenarios referring to the role of microbiota as either a cause or effect of cancer, the differences in sample collection and processing, methodological differences in sequencing, and variations in recruited patient groups, along with the distinct clinical and histopathological parameters, underrepresentation of some geographical regions, and patient-oriented studies only with few inconsistent and/or contradicting in vitro results, could ultimately make the generalization of the role of microbiota in health and disease difficult in a broader context. Therefore, further studies addressing all of the noted pitfalls are necessary, particularly in developing countries including Ethiopia to improve BC care. 

### 4.4. Why Shannon Diversity Was Not Different between NAT and Tumor Tissues

In this study, Shannon diversity or richness of microbial taxa was not found to be significantly different between NAT and tumor tissues; however, microbial evenness defined by Simpson’s index was slightly significant between the two groups. In support of our study, a previous study showed that adjacent normal paired breast tissue had a higher microbial diversity and richness than normal and tumor tissues [12]. Additionally, a previous study revealed that alpha diversity was significantly higher in normal compared to tumor samples where in unweighted UniFrac measures, breast tumor samples clustered distinctly from normal samples (R^2^ = 0.130; *p* = 0.01) [53]. One study from Ghana revealed that alpha diversity was strongly and inversely associated with BC by tumor stage and molecular subtype [66]. In other studies, it was shown that BC patients had statistically significantly altered microbiota composition (beta diversity) and lower alpha diversity compared with healthy patients [59] or breast tissues [12,67], whereas another study reported that there was no difference in bacterial communities between tumor tissue and NAT [22], which may be explained by sample retrieval approaches [26,50]. Since we did not include breast tissue from women without BC, such a discrepancy in the relative alpha diversity between normal tissue and tumor samples is anticipated. Such a scenario will strengthen a well-articulated future study that includes diverse samples and specific patient groups. 

### 4.5. Limitation of Our Study

Our findings have paved a way to understanding the composition of breast microbiota in Ethiopian BC patients. Despite the plethora of microbiome studies in developed countries, it has been shown that African populations are under-represented, and there is an acute demand for studies related to the microbiome among African populations [68]. Therefore, taking into account context-specific factors and considerable limited evidence, microbiome studies must be replicated in Africa to extend our understanding of BC and to identify potential biomarkers used for prognosis and predicting therapeutic response. We believe that having a larger sample size consisting of fresh frozen breast tissues from healthy controls (without BC) and BC patients with benign and malignant tumors might assist in drawing conclusions as to whether with microbial differences are a consequence or a cause of BC. Additional patient information on lifestyle factors, such as diet and environmental exposures, are also critical components for understanding the role of microbial dysbiosis on the breast microenvironment. 

## 5. Conclusions

In summary, the current study, which utilized fresh frozen breast tumor tissues and NAT from the same women, reported for the first time a unique microbial signature that correlates with prognostic features, including stage, IHC status, and PAM50 intrinsic subtypes, among Ethiopian women with BC. We identified 14 microbiota genera in breast tumor tissues that were distinct from NAT tissues. *Burkholderia* most strongly correlated with aggressive triple negative (TNBC) and basal-like breast tumors, whereas *Alkanindiges*, *Anoxybacillus*, *Leifsonia*, and *Exiguobacterium* most strongly correlated with HER2-E tumors. Luminal A and luminal B tumors also correlated with *Anoxybacillus* but not as strongly as HER2-E tumors. A relatively higher abundance of the genus *Citrobacter* most significantly correlated with advanced-stage breast tumors compared to early-stage tumors. This is the first study to report an association between breast microbial dysbiosis and clinicopathological factors in Ethiopian women. Our findings encourage further precise characterization of local microbes in BC patients in Ethiopia for drug discovery and targeted microbial-based therapeutics, thus improving the prognosis and quality of life of BC patients. A future epidemiological study taking into account sample size, more controlled environmental conditions, high throughput metagenomics, and follow-up data is essential to strengthen conclusions related to clinical outcomes.

## Figures and Tables

**Table 1:** IHC analysis with specific antibodies directed against ER, PR, HER2 and Ki-67.

Protein	Clone	Manufacturer	Host	Dilution
ER	Clone Ab-11	Thermo Scientific; cat. no MS-354-P1 (Waltham, MA, USA)	mouse	1:150
PR	Clone PgR 636	DAKO; cat. no M3569 (Santa Clara, CA, USA)	mouse	1:100
HER2	Clone DG44	DAKO; cat. no SK001	rabbit	RTU
Ki-67	Clone SP6	Thermo Scientific; cat. no RM-9106-S	mouse	1:250

**Table 2:** Patient and tumor characteristics.

Variable	Frequency (%)
**Mean age (yrs ± SE)**	46 ± 1.92
Minimum	27
Maximum	83
**Menopausal status**	
Pre	30 (60)
Post	17 (34)
Unknown	3 (6)
**UICC stage**	
Early (0–2A)	25 (50)
Advanced (2B–4)	17 (34)
Unknown	8 (16)
**Grade**	
1	0 (0)
2	24 (48)
3	26 (52)
**IHC type**	
HR+/HER2−	30 (60)
HR+/HER2+	14 (28)
HR−/HER2+	3 (6)
HR−/HER2−	3 (6)
**ER status (IHC)**	
Positive (**≥1%**)	44 (88)
Negative (<1%)	6 (12)
**PR status (IHC)**	
Positive (**≥1%**)	29 (58)
Negative (<1%)	21 (42)
**HER2 status (IHC)**	
Positive	17 (34)
Negative	33 (66)
**Ki-67 proliferation index (IHC)**	
Low (≥20%)	16 (32)
High (<20%)	34 (68)
**Intrinsic subtype**	
Luminal A	13 (26)
Luminal B	7 (17)
HER2-E	7 (14)
Basal-like	5 (10)
Unknown	18 (36)

## Data Availability

All relevant datasets generated and/or analyzed during the current study are included in this article, and we may provide additional de-identifiable data upon request.

## Supplementary Materials

The following supporting information can be downloaded at: https://www.mdpi.com/article/10.3390/cancers15194893/s1, Figure S1: Graphs show rarefaction curves from human breast tissue samples. (A) Rarefaction curve relating the number of sequencing reads compared to the number of amplicon sequence variants (ASVs). (B) Rarefaction curve relating the number of ASVs compared to the number of microbial genera. Figure S2: Bar plots illustrating the relative abundances of microbiota that differ between NAT and breast tumor tissues by (A) phylum, (B) class, and (C) family taxonomic levels. The unfilled portions of the bar plots represent lower-abundance taxa; Table S1: Significant genera by paired Wilcoxon signed-rank test in paired tumor relative to normal adjacent tissues; Table S2: Significant genera in tumors according to IHC status (linear model); Table S3: Significant genera in tumors according to PAM50 (linear model); Table S4: Significant genera that differ between advanced vs. early-stage tumors.
