# Supplementary material for: Human Breast Tissue Microbiota Reveals Unique Microbial Signatures that Correlate with Prognostic Features in Adult Ethiopian Women with Breast Cancer
Source: Cancers (Basel). 2023 Oct 9;15(19):4893. doi: 10.3390/cancers15194893 (PMC10571711; doi:10.3390/cancers15194893)
Supplement: Supplementary file 1 [file cancers-15-04893-s001.zip › cancers-2633564-supplementary.pdf]

Human breast tissue microbiota reveals unique microbial signatures that correlate with prognostic features in adult Ethiopian women with breast cancer

Zelalem Desalegn[1,2], Alana Smith[3], Meron Yohannes[1,2,4], Xueyuan Cao[5], Endale Anberber[6], Yonas Bekurestion[7], Matewos Assefa[8], Marcus Bauer[9], Martina Vetter[10], Eva Johanna Kantelhardt[2,10,11], Tamrat Abebe[1,2*±], and Athena Starlard-Davenport[3*±]

[1] Department of Microbiology, Immunology, and Parasitology, School of Medicine, Addis Ababa University, Addis Ababa 9086, Ethiopia; zelalem.desalegn@aau.edu.et; tzollove@gmail.com
[2] Global Health Working Group, Martin Luther University Halle-Wittenberg, Halle (Saale) 06097, Germany
[3] Department of Genetics, Genomics and Informatics, College of Medicine, University of Tennessee Health Science Center, Memphis, TN 38163, USA; astarlar@uthsc.edu; aantoin1@uthsc.edu
[4] School of Medical Laboratory Sciences, Addis Ababa University, Addis Ababa 9086, Ethiopia; meritijo@gmail.com; meron.yohannes@aau.edu.et
[5] Department of Health Promotion and Disease Prevention, College of Nursing, University of Tennessee Health Science Center, Memphis, TN 38163, USA; xcao12@uthsc.edu
[6] School of Medicine, Department of Surgery, Addis Ababa University, Addis Ababa 9086, Ethiopia; endale.anberber@gmail.com
[7] School of Medicine, Department of Pathology, Addis Ababa University, Addis Ababa 9086, Ethiopia; yonasbtsion@yahoo.com; yonas.bekuretsion@aau.edu.et
[8] School of Medicine, Department of Oncology, Addis Ababa University, Addis Ababa 9086, Ethiopia; mathewosassefa80@hotmail.com
[9] Institute of Pathology, Martin Luther University Halle-Wittenberg, Halle (Saale) 06097, Germany; marcus.bauer@uk-halle.de
[10] Department of Gynecology, Martin Luther University Halle-Wittenberg, Halle (Saale) 06097, Germany; eva.kantelhardt@uk-halle.de
[11] Institute of Medical Epidemiology, Biostatistics, and Informatics, Martin Luther University Halle-Wittenberg, Halle (Saale) 06097, Germany; eva.kantelhardt@uk-halle.de
* Correspondence: astarlar@uthsc.edu; Tel.: +1 901-448-3085; tamrat.abebe@aau.edu.et; Tel:+251911447227
±These authors contributed equally to this work

Citation: To be added by editorial staff during production.

Academic Editor: Firstname Lastname

Received: date
Revised: date
Accepted: date
Published: date

Copyright: © 2023 by the authors. Submitted for possible open access publication under the terms and conditions of the Creative Commons Attribution (CC BY) license (https://creativecommons.org/licenses/by/4.0/).

Supplemental Tables and Figures

Table S1: Significant genera by paired Wilcoxon signed-rank in paired tumor relative to normal adjacent tumor tissues

Genus	MedianIQR	P-value	BH95
Sphingobium	-0.347 (-2.134, 0)	0.0001	0.0105
Anaerococcus	-0.159 (-1.475, 0)	0.0001	0.0105
Corynebacterium	-0.693 (-2.359, 0.484)	0.0012	0.0632
Delftia	0 (-1.746, 0)	0.0031	0.1225
Enhydrobacter	0 (-1.314, 0)	0.0065	0.187
Cloacibacterium	-0.784 (-1.708, 0)	0.0071	0.187
Polaromonas	0 (0, 0)	0.0103	0.214
Peptoniphilus	0 (-1.314, 0)	0.0134	0.214
Varibaculum	0 (0, 0)	0.0135	0.214
Finegoldia	0 (-1.386, 0)	0.0142	0.214
Staphylococcus	-0.656 (-2.223, 0.444)	0.0149	0.214
Micrococcus	0 (-0.693, 0)	0.0175	0.2261
Prevotella	-0.347 (-1.365, 0)	0.0186	0.2261
Variovorax	0 (0, 0)	0.0239	0.2697
Coprobacter	0 (0, 0)	0.0316	0.3329
Brevibacterium	0 (0, 0)	0.0343	0.3387
Rhizobacter	0 (0, 0)	0.0411	0.3786
Bergeyella	0 (0, 0)	0.0477	0.3786

Abbreviations: BH: q value of false discovery rate using Benjamin Hochberg 1995 method; IQR: Interquartile Range
$P<0.05$ is statistically significant

Table S2: Significant genera in tumors according to IHC status (linear model)

Genus	Level	Estimate	SE	P-value	P
Exiguobacterium	HR+HER2-	-0.366	0.1	0.0006	0.007
	HR+HER2+	-0.317	0.105	0.0041	
	TNBC	-0.366	0.135	0.0092	
Rhodopseudomonas	HR+HER2-	-0.647	0.588	0.2773	0.0376
	HR+HER2+	0.282	0.618	0.6509	
	TNBC	-0.157	0.793	0.8444	
Varibaculum	HR+HER2-	-0.561	0.162	0.0012	0.0086
	HR+HER2+	-0.597	0.171	0.001	
	TNBC	-0.597	0.219	0.009	
Polaromonas	HR+HER2-	0	0.08	1	5.00E-04
	HR+HER2+	0	0.084	1	
	TNBC	0.366	0.108	0.0014	
Aeromonas	HR+HER2-	0.023	0.151	0.8792	0.0047
	HR+HER2+	0.099	0.159	0.536	
	TNBC	0.597	0.204	0.0053	
Romboutsia	HR+HER2-	0.116	0.218	0.5993	0.0226
	HR+HER2+	0.05	0.229	0.8301	
	TNBC	0.768	0.294	0.0123	
Pedobacter	HR+HER2-	0.037	0.272	0.8934	0.0198
	HR+HER2+	0.128	0.286	0.6562	
	TNBC	0.924	0.367	0.0152	
Leifsonia	HR+HER2-	-0.67	0.163	0.0002	0.0016
	HR+HER2+	-0.693	0.172	0.0002	
	TNBC	-0.693	0.22	0.0029	
Cupriavidus	HR+HER2-	0.046	0.145	0.7508	0.0086
	HR+HER2+	0	0.152	1	
	TNBC	0.536	0.195	0.0085	

NOTE: HR-HER2- tumors serve as the reference group
Abbreviations: SE: Standard Error; P<0.05 is statistically significant

Table S3: Significant genera in tumors according to PAM50 (linear model)

Genus	Level	Estimate	SE	P-value	P
Exiguobacterium	luminal A	-0.022	0.055	0.6857	0.0097
	HER2-E	0.344	0.1	0.0013	
	basal-like	-0.022	0.1	0.8248	
Bifidobacterium	luminal A	0.208	0.245	0.3998	0.0382
	HER2-E	-0.741	0.448	0.1049	
	basal-like	1.007	0.448	0.0293	
Varibaculum	luminal A	-0.035	0.089	0.6911	0.0087
	HER2-E	0.562	0.162	0.0012	
	basal-like	-0.035	0.162	0.828	
Polaromonas	luminal A	0	0.044	1	5.00E-04
	HER2-E	0	0.08	1	
	basal-like	0.366	0.08	0	
Aeromonas	luminal A	-0.067	0.083	0.4214	0.0052
	HER2-E	-0.067	0.151	0.6596	
	basal-like	0.53	0.151	0.001	
Romboutsia	luminal A	0.093	0.119	0.4383	0.0198
	HER2-E	-0.067	0.217	0.759	
	basal-like	0.7	0.217	0.0023	
Pedobacter	luminal A	0.222	0.145	0.1329	0.0082
	HER2-E	0	0.266	1	
	basal-like	0.924	0.266	0.0011	
Leifsonia	luminal A	-0.022	0.089	0.8031	0.0016
	HER2-E	0.671	0.163	0.0002	
	basal-like	-0.022	0.163	0.8916	
Cupriavidus	luminal A	0.031	0.079	0.6973	0.0095
	HER2-E	-0.022	0.145	0.8779	
	basal-like	0.514	0.145	0.0009	

NOTE: Luminal B tumors serve as the reference group
Abbreviations: SE: Standard Error; P<0.05 is statistically significant

Table S4: Significant genera that differ between advanced vs early stage tumors

Genus	Estimate*	SE	P-value
Stenotrophomonas	1.51	0.597	0.0155
Corynebacterium	1.122	0.554	0.0494
Prevotella	1.11	0.475	0.0244
Actinomyces	0.814	0.338	0.0207
Streptococcus	0.668	0.317	0.0416
Anaerococcus	0.618	0.303	0.0484
Citrobacter	0.571	0.196	0.0059
Dermacoccus	0.252	0.103	0.0192
Blastococcus	0.194	0.086	0.0294

*Estimate: Early staged tumors serve as the reference group
Abbreviations: SE: Standard Error; P<0.05 is statistically significant

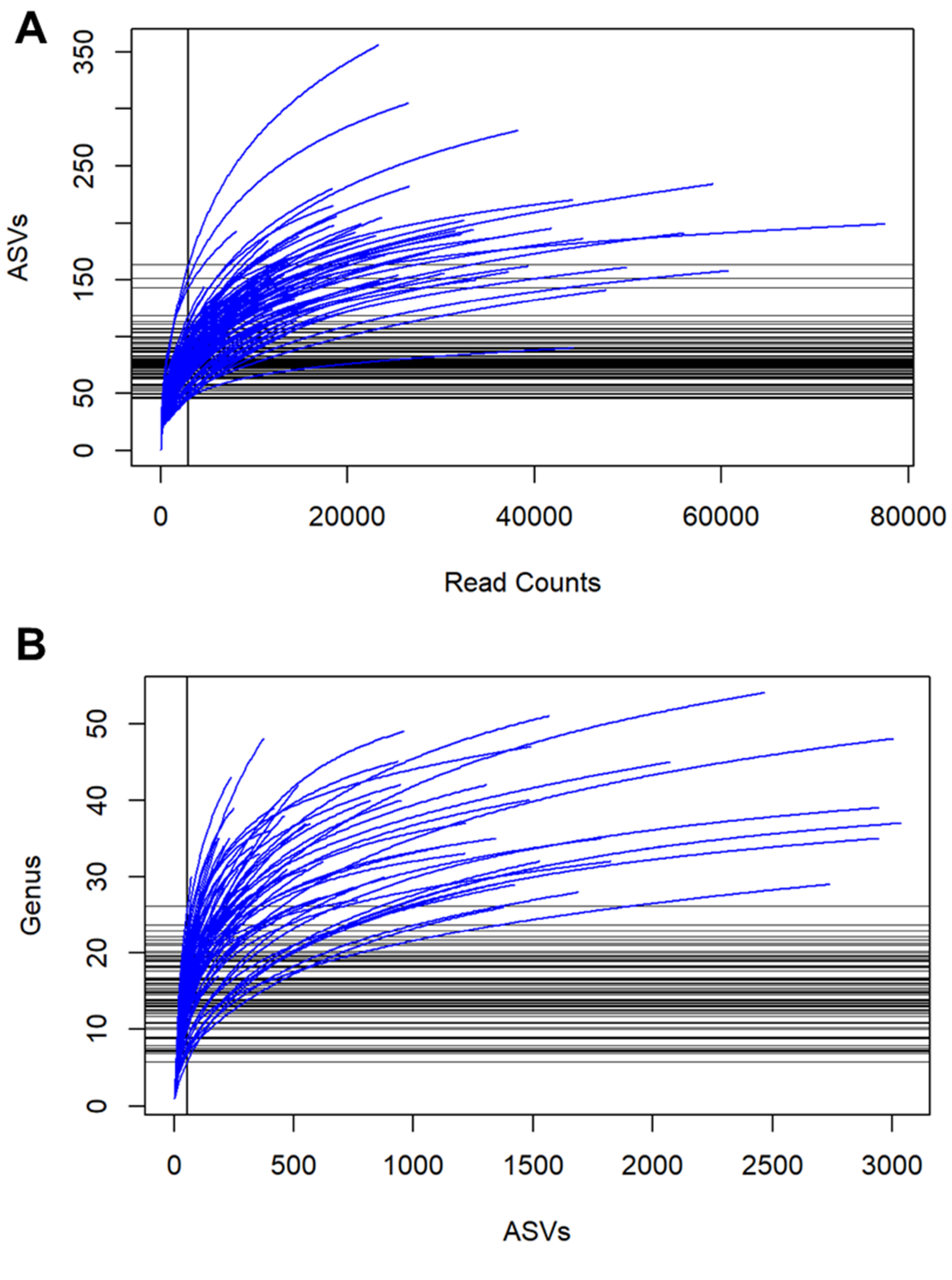

Figure S1. Graphs show rarefaction curves from human breast tissue samples. (A) Rarefaction curve relating the number of sequencing reads compared to the number of amplicon sequence variants (ASVs). **(B)** Rarefaction curve relating the number of ASVs compared to the number of microbial genera.

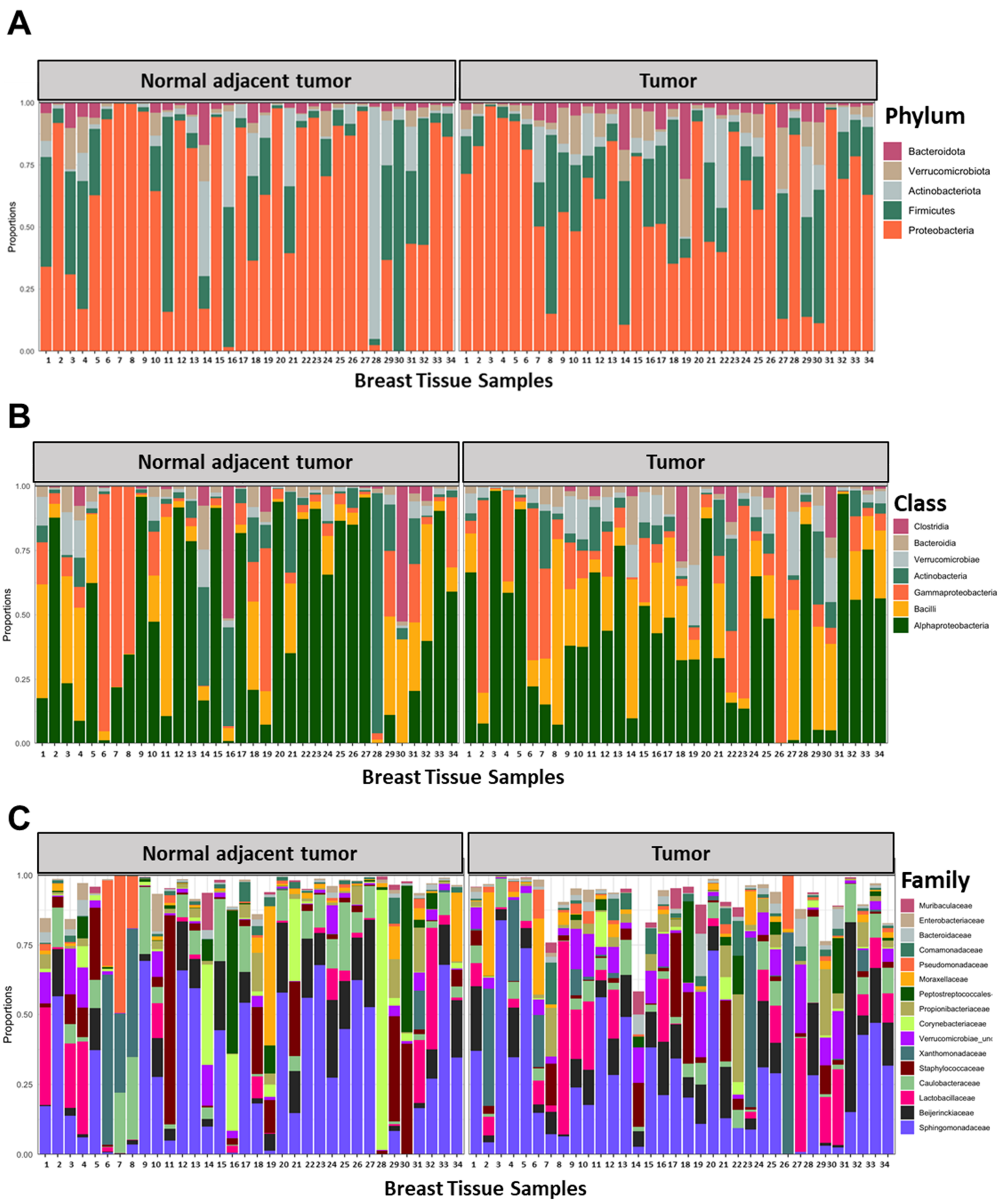

Figure S2. Bar plots illustrating the relative abundances of microbiota that differ between NAT and breast tumor tissues by (A) phylum, (B) class, and (C) family taxonomic levels. The unfilled portion of the bar plots represent lower-abundance taxa.
